# Comparative performance of BD-BACTEC^®^ Mycosis IC/F versus standard aerobic and anaerobic bottles in simulated fungemia and mixed bloodstream infections

**DOI:** 10.1590/S1678-9946202567064

**Published:** 2025-10-03

**Authors:** Juliene Carla Endo Borges, Lumena Pereira Machado Siqueira, Vera Lucia Teixeira de Freitas, Vítor Falcão de Oliveira, Adriana Satie Gonçalves Kono Magri, Afonso Rafael da Silva, Evangelina da Motta Pacheco Alves de Araujo, Ana Paula Cury, Marcello Mihailenko Chaves Magri

**Affiliations:** 1 Universidade de São Paulo, Faculdade de Medicina, Hospital das Clínicas, Divisão de Laboratório Central, Seção de Microbiologia, São Paulo, São Paulo, Brazil; 2 Universidade de São Paulo, Faculdade de Medicina, Hospital das Clínicas, Laboratório de Investigação Médica (LIM-48), São Paulo, São Paulo, Brazil; 3 Universidade de São Paulo, Faculdade de Medicina, Hospital das Clínicas, Divisão de Clínica de Moléstias Infecciosas e Parasitárias, São Paulo, São Paulo, Brazil

**Keywords:** *Candida* spp, Bloodstream infections, Fungal detection, BD BACTEC™, Mycosis IC/F, Polymicrobial infection, Blood culture

## Abstract

Candidemia is a leading cause of bloodstream infection–associated morbidity and mortality, particularly among critically ill patients. Time to detection (TTD) is crucial, but standard blood culture systems often fail to recover yeasts, filamentous fungi, or identify fungi in polymicrobial infections. The BD BACTEC^™^ Mycosis IC/F bottle was designed to improve fungal detection, yet comparative performance data are limited. This study aimed to compare the detection rate and TTD of BD BACTEC^™^ Mycosis IC/F with those of standard BD BACTEC^™^ Plus Aerobic/F and BD BACTEC^™^ Plus Anaerobic/F bottles using simulated models of fungemia and mixed fungal–bacterial bloodstream infections. This *in vitro* study included 333 blood culture bottles inoculated with *Candida* spp., *Cryptococcus neoformans, Trichosporon asahii, Fusarium* spp., and *Aspergillus terreus*, as well as bottles simulating coinfections with multidrug-resistant Gram-negative bacteria. All bottles were incubated in the BD BACTEC™ FX system for up to 14 days. BD BACTEC^™^ Mycosis IC/F achieved 100% detection for *Candida* spp., outperforming BD BACTEC^™^ Plus Anaerobic/F (58.5%) and matching BD BACTEC^™^ Plus Aerobic/F. It showed shorter TTDs for *Nakaseomyces glabratus, Candidozyma haemuli, Meyerozyma guilliermondii,* and molds. In mixed infections, BD BACTEC^™^ Mycosis IC/F provided better fungal recovery, especially at low inocula, although recovery was impaired when coinoculated with carbapenemase-producing bacteria. In conclusion, BD BACTEC^™^ Mycosis IC/F improved fungal detection and recovery compared with standard bottles, including in polymicrobial settings. Its use may enhance diagnostic yield in suspected fungemia, though cost and limited availability may limit widespread adoption.

## INTRODUCTION

Candidemia, defined as the isolation of *Candida* spp. in blood cultures, is one of the most frequent invasive fungal infections, particularly among critically ill patients, and is associated with high mortality rates, often exceeding 50%^1-3^. It remains the most common fungal bloodstream infection in hospitalized patients and ranks as the fourth most frequent healthcare-associated infection in intensive care units (ICUs)^4^. *Candida* spp. comprises over 200 species, with *C. albicans, Nakaseomyces glabratus* (*C. glabrata*)*, C. tropicalis*, the *C. parapsilosis* complex, *Pichia kudriavzevii* (*C. krusei*), *Candidozyma auris* (*C. auris*)*, Candidozyma haemuli* (*C. haemulonii*), and *Meyerozyma guilliermondii* (*C. guilliermondii*) accounting for over 90% of clinical isolates^5,6^. While *C. albicans* continues to be the most commonly isolated species, recent epidemiological shifts have highlighted the increasing prevalence of non-albicans *Candida* species, as well as other yeast-like fungi and filamentous fungi such as *Fusarium* spp. and *Aspergillus* spp., which often exhibit greater antifungal resistance and are associated with poorer clinical outcomes^1,7,8^.

Numerous risk factors contribute to the development of candidemia, including broad-spectrum antibiotic use, immunosuppression, organ transplantation, abdominal surgery, central venous catheterization, total parenteral nutrition, and prematurity^1,7^. The COVID-19 pandemic has emerged as an additional risk factor, with studies reporting increased incidence, earlier onset, and higher mortality in co-infected patients^3,9^. Regional differences in incidence and outcomes are also evident, with Latin American countries, including Brazil, reporting higher candidemia rates than North America or Europe^1,10-14^.

Despite being the current gold standard, blood culture for diagnosing *Candida* spp. bloodstream infections suffers from suboptimal sensitivity, often below 50%, and delayed positivity, particularly in the context of co-infections or prior antifungal exposure^1,5,15^. Automated culture systems such as BD-BACTEC^®^ and BACT/ALERT^®^ have improved detection rates and turnaround times^16-21^, but species like *N. glabratus* may still require up to 80 h for detection^17^. Specific media such as BD-BACTEC^®^ BD BACTEC^™^ Mycosis IC/F have been developed to enhance fungal recovery by inhibiting bacterial overgrowth and reducing time to detection, showing promise in studies using controlled inoculum of *Candida* species^21^.

This pilot study aims to evaluate the diagnostic performance of the BD BACTEC^™^ Mycosis IC/F bottle in comparison with standard aerobic and anaerobic media (BD BACTEC^™^ Plus Aerobic/F and BD BACTEC^™^ Plus Anaerobic/F) using simulated fungemia models. The study analyzes positivity rates, time to detection (TTD), and fungal recovery in the presence of mixed infections. By employing standardized inoculum and experimental conditions, this research seeks to provide data that may improve diagnostic strategies for candidemia and guide the selection of appropriate blood culture media in clinical laboratories.

## MATERIALS AND METHODS

### Ethics

The study was conducted in accordance with the Declaration of Helsinki. Ethical approval for the study was obtained from the institution’s research ethics committee (protocol Nº 79796324.8.0000.0068).

### Study isolates

A total of 21 fungal isolates were included, comprising both clinical and reference strains (ATCC), representing the most frequently identified species and clinically important emerging pathogens in blood cultures. These included: *C. albicans* (clinical and ATCC 14053), *C. auris* (CBS 12766), *N. glabratus* (clinical and ATCC MYA 2950), *M. guilliermondii* (clinical and ATCC 168), *C. haemuli* (clinical and CBS 5149), *P. kudriavzevii* (clinical and ATCC 6258), *C. tropicalis* (clinical and ATCC 750), *Trichosporon asahii* (clinical and CBS 5599), *Cryptococcus neoformans* (clinical and ATCC 6901-VNIV), *Neocosmospora solani* formerly *Fusarium solani* (clinical and CBS 124633), and *Aspergillus terreus* (clinical and CBS 11737). Isolates were obtained from clinical samples processed at the Microbiology Laboratory of the Hospital das Clinicas, University of Sao Paulo Medical School (HCFMUSP), or from reference culture collections.

To simulate coinfections, bacterial strains, including both susceptible and resistant phenotypes, were also included: *Staphylococcus epidermidis* ATCC 12228, methicillin-resistant *Staphylococcus aureus* (MRSA) ATCC BAA 1026, *Acinetobacter baumannii* OXA-23, *Pseudomonas aeruginosa* ATCC 2753, *Klebsiella pneumoniae* KPC ATCC BAA 1705, *P. aeruginosa* (clinical isolates producing IMP or co-producing VIM and KPC), and *K. pneumoniae* producing NDM. All strains were subcultured from −80 °C stocks to ensure viability and purity. *Candida* spp. were cultured on CHROMagar^®^ *Candida* (Plastlabor) at 37 °C for 18–24 h. Filamentous fungi were cultured on potato dextrose agar (PDA, ThermoFisher) at 30 °C for 72 h. Bacteria were grown on blood and MacConkey agar.

### Blood culture bottles

A total of three types of BD BACTEC™ bottles were analyzed: BD BACTEC^™^ Mycosis IC/F, BD BACTEC^™^ Plus Aerobic/F, and BD BACTEC^™^ Plus Anaerobic/F (Becton Dickinson), totaling 111 bottles of each type. Aerobic and anaerobic bottles contain resins that neutralize antimicrobial agents, while BD BACTEC^™^ Mycosis IC/F is a selective fungal medium supplemented with chloramphenicol and tobramycin to inhibit bacterial growth.

### Simulated fungemia

Suspensions of *Candida* spp. were prepared in sterile 0.9% NaCl to a 0.5 McFarland standard (approx. 1.5 × 10^6^ CFU/mL). Serial 1:10, 1:100, and 1:1000 dilutions were performed to yield 10^5^, 10^4^, and 10^3^ CFU/mL. Then, 10 mL of each dilution were inoculated into the three blood culture bottle types in triplicate for each fungal strain. Filamentous fungi were incubated on Potato Dextrose Agar (Thermo Fisher Scientific, Waltham, MA, USA) to promote sporulation. After sufficient maturation, conidial suspensions were filtered using a 40 µm cell strainer (JetBiofil CSS 013040) to remove hyphal fragments. Conidia were counted using a Neubauer chamber and diluted to a final concentration of 1–2.5 × 10^5^ conidia/mL, as recommended by BrCAST (Brazilian Committee on Antimicrobial Susceptibility Testing). Serial dilutions (1:10, 1:100, 1:1000) were then inoculated (10 mL) into each of the three blood culture bottle types.

### Simulated mixed candidemia–bacteremia

Mixed suspensions were prepared by combining *C. albicans or N. glabratus* with one bacterial isolate, each adjusted to 0.5 McFarland (1.5 × 10^8^ CFU/mL). The final inoculum was prepared by mixing equal volumes (5 mL each) of fungal and bacterial suspensions, followed by serial dilutions (1:10, 1:100, 1:1000). Then, 10 mL of each dilution were inoculated into each type of blood culture bottle.

### Incubation and Contamination Control

Blood culture bottles were incubated in the BD BACTEC^™^ FX Automated Blood Culture System (Becton, Dickinson and Company, Sparks, MD, USA). Following the manufacturer’s instructions, BD BACTEC^™^ Plus Aerobic/F and BD BACTEC^™^ Plus Anaerobic/F bottles were incubated for 5 days, whereas BD BACTEC^™^ Mycosis IC/F bottles were incubated for 14 days. Bottles without a positive signal at the end of the respective incubation period were considered negative. All inoculations were performed under aseptic conditions in a laminar flow hood using sterile, single-use materials. To monitor for potential contamination, negative control bottles containing only sterile saline (without fungal or bacterial inoculum) were included in each experimental batch and incubated alongside test bottles under identical conditions.

Upon positivity or at the end of incubation, a standardized aliquot from each bottle was plated onto selective media based on the suspected organism: CHROMagar^™^ *Candida* Medium (CHROMagar, Paris, France), Potato Dextrose Agar (Thermo Fisher Scientific, Waltham, MA, USA), or CHROMagar^™^ Orientation CPS^®^ Medium (Plastlabor, Rio de Janeiro, Brazil). For coinfection simulations, Gram staining was also performed directly from the culture broth. All cultures were incubated at either 30 °C or 37 °C, depending on the organism. Specifically, all bacterial strains (*K. pneumoniae, A. baumannii, and P. aeruginosa*) were incubated at 35 ± 2 °C for 18–24 h on Mueller-Hinton agar prior to suspension preparation. Time to detection (TTD) was recorded for each bottle in hours and minutes, and automated positivity curves were documented for subsequent analysis.

### Data analysis

Descriptive and comparative statistical analyses were performed on TTD values across BD BACTEC^™^ Mycosis IC/F, BD BACTEC^™^ Plus Aerobic/F, and BD BACTEC^™^ Plus Anaerobic/F bottles. Means and medians were calculated for each dilution and strain type (clinical or ATCC). Relative differences in detection time between bottle types were expressed as percentage variation, calculated using the following formulas:


$$\mathrm{Variation}(\%)=\frac{TTD_{\text{Mycosis }}-TTD_{\text{Aerobic }}}{TDD_{\text{Anaerobic }}}\times 100$$

$$\mathrm{Variation}(\%)=\frac{TTD_{\text{Mycosis }}-TTD_{\text{Anaerobic }}}{TDD_{\text{Aerobic }}}\times 100$$

Bar graphs were generated to illustrate detection time comparisons and percentage variations between bottle types for each fungal strain.

## RESULTS

A total of 333 blood culture bottles were incubated, comprising 111 BD BACTEC^™^ Mycosis IC/F, 111 BD BACTEC^™^ Plus Aerobic/F, and 111 BD BACTEC^™^ Plus Anaerobic/F bottles. Both BD BACTEC^™^ Mycosis IC/F and BD BACTEC^™^ Plus Aerobic/F bottles achieved 100% positivity (111/111), whereas BD BACTEC^™^ Plus Anaerobic/F bottles demonstrated a significantly lower positivity rate of 58.5% (65/111). Most positive results from the anaerobic bottles were associated with *Candida* spp. isolates. In contrast, filamentous fungi, coinfecting bacterial strains, and non-*Candida* yeasts exhibited minimal growth under anaerobic conditions.

For BD BACTEC^™^ Mycosis IC/F bottles, the mean time to detection (TTD) was approximately 24 h (range: 5-165 h). For BD BACTEC^™^ Plus Aerobic/F bottles, the mean TTD was about 20 h (range: 5–89 h). Among the positive BD BACTEC^™^ Plus Anaerobic/F bottles, the mean TTD was 23 h (range: 5.6–91.7 h).

### Detection of Candida spp.

All *Candida* spp. isolates were successfully detected within 58 h by both BD BACTEC^™^ Mycosis IC/F and BD BACTEC^™^ Plus Aerobic/F bottles at all dilutions. In contrast, BD BACTEC^™^ Plus Anaerobic/F bottles showed limited sensitivity, detecting only 53.8% (21/39) of samples, with failures concentrated in *C. auris, C. haemuli*, and *P. kudriavzevii* (clinical strain at 1:100 and 1:1000), as well as *C. albicans* and *M. guilliermondii* at the lowest inoculum (Table 1). For *C. albicans*, both BD BACTEC^™^ Mycosis IC/F and BD BACTEC^™^ Plus Aerobic/F achieved 100% positivity. The shortest TTDs were found for BD BACTEC^™^ Mycosis IC/F (8 h 09 min for the clinical strain, 8 h 16 min for the ATCC strain at 1:10). Percentage variation between BD BACTEC^™^ Mycosis IC/F and BD BACTEC™ Plus Aerobic/F was minimal (0.11 to 0.8), whereas BD BACTEC^™^ Mycosis IC/F detected growth 1.26 to 4.29 times faster than BD BACTEC™ Plus Anaerobic/F. (Supplementary Figure S1A).

**Table 1:** Time to detection (TTD) in hours in BD BACTEC™ Mycosis IC/F, BD BACTEC™ Plus Aerobic/F and BD BACTEC™ Plus Anaerobic/F blood culture bottles

Microorganism		TTD (h) 1:10				TTD (h) 1:100				TTD (h) 1:1000		
		MYC	AER	ANA		MYC	AER	ANA		MYC	AER	ANA
*C. albicans*	ATCC	8 h 16 min	9 h 09 min	18 h 43 min		12 h 55 min	11 h 51 min	2 d 20 h 17 min		15 h 58 min	14 h 58 min	Negative
	Clinical	8 h 09min	8 h 48 min	12 h 09 min		11 h 51 min	12 h 31 min	16 h 27 min		23 h 30 min	1 d 1 h 31 min	Negative
*N. glabratus*	ATCC	5 h 03 min	7 h 35 min	12 h 19 min		10 h 36 min	11 h 38 min	15 h 40 min		13 h 15 min	16 h 14 min	23 h 15 min
	Clinical	6 h 12 min	7 h 52 min	10 h 43 min		9 h 23 min	12 h 27 min	15 h 48 min		11 h 11 min	14 h 15 min	21 h 44 min
*C. tropicalis*	ATCC	7 h 30 min	6 h 28 min	6 h 26 min		11 h 39 min	10 h 40 min	17 h 30 min		12 h 37 min	13 h 51 min	18 h 27 min
	Clinical	7 h 07 min	5 h 23 min	5 h 36 min		10 h 37 min	8 h 05 min	10 h 06 min		15 h 08 min	10 h 43 min	16 h 05 min
*P. kudriavzevii*	ATCC	7 h 47 min	9 h 46 min	9 h 43 min		10 h 37 min	11 h 52 min	14 h 33 min		12 h 57 min	16 h 48 min	22 h 34 min
	Clinical	8 h 48 min	7 h 45 min	18 h 43 min		13 h 24 min	10 h 42 min	Negative		19 h 59 min	14 h 00 min	Negative
*C. haemuli*	ATCC	11 h 34 min	14 h 06 min	Negative		16 h 04 min	1 d 6 h 06 min	Negative		1 d 2 h 36 min	2 d 10 h 32 min	Negative
	Clinical	10 h 28 min	11 h 46 min	Negative		14 h 41 min	18 h 39 min	Negative		19 h 40 min	1 d 3 h 39 min	Negative
*M. guilliermondii*	ATCC	10 h 37 min	11 h 21 min	3 d 4 h 40 min		18 h 36 min	20 h 39 min	Negative		18 h 38 min	21 h 43 min	Negative
	Clinical	9 h 24 min	10 h 42 min	Negative		13 h 11 min	15 h 48 min	Negative		18 h 23 min	20 h 24 min	Negative
*C. auris*	ATCC	10 h 27 min	10 h 24 min	Negative		13 h 37 min	15 h 03 min	Negative		19 h 10 min	21 h 08 min	Negative
*C. neoformans*	ATCC	17 h 06 min	17 h 35 min	Negative		1 d 4 h 08 min	1 d 1 h 04 min	Negative		1 d 10 h 09 min	1 d 8 h 10 min	Negative
	Clinical	11 h 40 min	13 h 08 min	Negative		19 h 11 min	19 h 39 min	Negative		1 d 3 h 12 min	1 d 3 h 39 min	Negative
*T. asahii*	ATCC	10 h 37 min	9 h 17 min	Negative		20 h 46 min	12 h 16 min	Negative		1 d 1 h 44 min	16 h 13 min	Negative
	Clinical	13 h 16 min	9 h 57 min	3 d 19 h 42 min		21 h 49 min	12 h 46 min	Negative		1 d 8 h 15 min	16 h 43 min	Negative
*A. terreus*	ATCC	22 h 33 min	1 d 3 h 34 min	Negative		1 d 4 h 13 min	1 d 9 h 03 min	Negative		1 d 12 h 03 min	1 d 15 h 03 min	Negative
	Clinical	1 d 0 h 38 min	1 d 0 h 10 min	Negative		3 d 6 h 26 min	1 d 14 h 12 min	Negative		4 d 22 h 10 min	2 d 7 h 16 min	Negative
*N. solani*	ATCC	17 h 03 min	22 h 34 min	Negative		1 d 0 h 04 min	1 d 3 h 08 min	Negative		1 d 7 h 07 min	1 d 10 h 38 min	Negative
	Clinical	3 d 3 h 05 min	1 d 10 h 12 min	Negative		5 d 5 h 23 min	2 d 9 h 56 min	Negative		6 d 21 h 51 min	3 d 17 h 30 min	Negative

ATCC = Reference strain; Clinical = Clinical isolate obtained from the Microbiology Laboratory, Hospital das Clinicas, University of Sao Paulo Medical School.

*N. glabratus* was detected in all bottles and dilutions. BD BACTEC^™^ Mycosis IC/F provided the fastest TTDs (5 h 03 min for ATCC and 6 h 12 min for the clinical strain at 1:10). Variation between BD BACTEC^™^ Mycosis IC/F and BD BACTEC^™^ Plus Aerobic/F ranged from 0.07 to 0.4, whereas differences with BD BACTEC^™^ Plus Anaerobic/F reached up to 1.8-fold, especially at 1:1000 dilution (Supplementary Figure S1B). For *C. tropicalis*, BD BACTEC^™^ Plus Aerobic/F showed the shortest TTDs in most conditions (e.g., 6 h 28 min at 1:10 for ATCC), with BD BACTEC^™^ Mycosis IC/F being slightly slower (variation 1.05 to 1.3), and BD BACTEC^™^ Plus Anaerobic/F showing a prolonged TTDs (Supplementary Figure S1C).

Detection of *P. kudriavzevii* was most efficient with BD BACTEC^™^ Mycosis IC/F for the ATCC strain (7 h 47 min at 1:10). For the clinical isolate, BD BACTEC^™^ Plus Aerobic/F was faster than BD BACTEC^™^ Mycosis IC/F at all tested dilutions. TTD variation between BD BACTEC^™^ Mycosis IC/F and BD BACTEC^™^ Plus Aerobic/F ranged from 0.12 to 0.30, whereas BD BACTEC^™^ Plus Anaerobic/F lagged substantially (variation 0.25 to 1.13) and failed at higher dilutions (Supplementary Figure S1D). For *M. guilliermondii,* BD BACTEC^™^ Mycosis IC/F consistently provided shorter TTDs (e.g., 10 h 37 min at 1:10 for ATCC), followed by BD BACTEC^™^ Plus Aerobic/F. BD BACTEC^™^ Plus Anaerobic/F detected only one strain at 1:10, with TTD exceeding 72 h. Percentage variation confirmed the poorer performance of BD BACTEC^™^ Plus Anaerobic/F, particularly at 1:100 and 1:1000 (Supplementary Figure S2E).

*C. haemuli* was detected only by BD BACTEC^™^ Mycosis IC/F and BD BACTEC^™^ Plus Aerobic/F. For the ATCC strain, TTDs were 11 h 34 min and 14 h 06 min, respectively, with variations ranging from 1.1 to 2.2 across dilutions. BD BACTEC^™^ Plus Anaerobic/F failed entirely (Supplementary Figure S2F). Finally, *C. auris* ATCC was detected by both BD BACTEC^™^ Mycosis IC/F and BD BACTEC^™^ Plus Aerobic/F at 1:10 dilution (TTDs: 10 h 27 min and 10 h 24 min, respectively); however, BD BACTEC^™^ Mycosis IC/F detected the organism faster at 1:100 and 1:1000. BD BACTEC^™^ Plus Anaerobic/F failed to detect growth at all dilutions (Supplementary Figure S1G). Table 1 summarizes TTD data, and Supplementary Table S1 shows all results.

### Detection of T. asahii

Table 1 and Supplementary Figure S3 show the comparative time to detection (TTD) for *T. asahii* in BD BACTEC^™^ Mycosis IC/F, BD BACTEC^™^ Plus Aerobic/F, and BD BACTEC^™^ Plus Anaerobic/F bottles across three serial dilutions (1:10, 1:100, and 1:1000) for both ATCC and clinical strains. In both strains, BD BACTEC^™^ Plus Aerobic/F demonstrated the shortest TTDs at all dilutions. BD BACTEC^™^ Mycosis IC/F detected growth slightly later but consistently. BD BACTEC^™^ Plus Anaerobic/F either failed to detect growth or showed markedly delayed detection—particularly for the clinical strain at 1:10, in which positivity occurred only after nearly four days.

### Detection of C. neoformans

In contrast to the pattern observed with *T. asahii*, *C. neoformans* showed similar TTDs in BD BACTEC^™^ Mycosis IC/F and BD BACTEC^™^ Plus Aerobic/F bottles, with a slight advantage for BD BACTEC^™^ Mycosis IC/F across most dilutions. For the clinical strain, TTDs in BD BACTEC^™^ Mycosis IC/F were shorter than those observed for the ATCC strain. As with *T. asahii*, BD BACTEC^™^ Plus Anaerobic/F was ineffective for detecting *C. neoformans*, showing no positivity at any tested dilution (Table 1 and Supplementary Figure S3B).

### Detection of filamentous fungi: N. solani and A. terreus

Among the four fungal isolates belonging to *A. terreus* and *N. solani*, growth was observed in 24 out of 36 blood culture bottles. All BD BACTEC^™^ Mycosis IC/F and BD BACTEC^™^ Plus Aerobic/F bottles yielded positive results, corresponding to a positivity rate of 100%. For *A. terreus*, BD BACTEC^™^ Mycosis IC/F showed shorter TTD compared with BD BACTEC^™^ Plus Aerobic/F, particularly for the ATCC strain, across all tested dilutions. A similar pattern was observed for the ATCC strain of *N. solani*. However, for the clinical isolates of *A. terreus* and *N. solani*, growth occurred significantly faster in BD BACTEC^™^ Plus Aerobic/F bottles than in BD BACTEC^™^ Mycosis IC/F. Notably, however, only a single clinical strain was tested. BD BACTEC™ Plus Anaerobic/F bottles showed no growth for any of the organisms at any dilution (Table 1 and Supplementary Figure S4)*.*

### BD BACTEC™ mycosis IC/F compared to BD BACTEC™ Plus Aerobic/F bottles

The most pronounced differences in TTD between BD BACTEC^™^ Mycosis IC/F and BD BACTEC^™^ Plus Aerobic/F bottles, favoring the latter, were observed for *T. asahii* (both ATCC and clinical strains), with BD BACTEC^™^ Plus Aerobic/F demonstrating a mean detection time of 12.8 h compared to 20.7 h with BD BACTEC^™^ Mycosis IC/F, an approximate difference of 7.9 h. A similar pattern was observed for *C. tropicalis* (ATCC and clinical strains), in which the mean detection time was 9.2 h with BD BACTEC™ Plus Aerobic/F versus 10.7 h with BD BACTEC^™^ Mycosis IC/F (Table 1).

### Detection in simulated mixed candidemia and bacteremia

BD BACTEC^™^ Mycosis IC/F and BD BACTEC^™^ Plus Aerobic/F bottles demonstrated the best performance in detecting *C. albicans* and *N. glabratus* in the presence of various bacterial species, even with low fungal inoculum (1:1000). BD BACTEC^™^ Plus Anaerobic/F frequently failed to detect fungal growth in co-infection settings involving *N. glabratus* plus IMP or *N. glabratus* plus VIM/KPC, particularly at the 1:100 and 1:1000 dilutions. Notably, *C. albicans* co-infected with *P. aeruginosa* (IMP) or *K. pneumoniae* (NDM) was detected only by BD BACTEC^™^ Mycosis IC/F at 1:1000, underscoring the risk of false negatives with conventional bottles in the context of mixed infections (Table 2).

**Table 2:** Time to detection (TTD) in hours and minutes for simulated mixed candidemia and bacteremia in Mycosis IC/F, Aerobic/F, and Anaerobic/F blood culture bottles

Microorganism	Bacteria	TTD (h) 1:10				TTD (h) 1:100				TTD (h) 1:1000		
		MYC	AER	ANA		MYC	AER	ANA		MYC	AER	ANA
*C. albicans*	KPC	9 h 55 min	4 h 06 min	3 h 53 min		15 h 37 min	6 h 17 min	5 h 12 min		19 h 37 min	10 h 18 min	6 h 00 min
*C. albicans*	MRSA	10 h 39 min	5 h 26 min	6 h 29 min		15 h 36 min	8 h 29 min	9 h 01 min		19 h 37 min	11 h 10 min	11 h 31 min
*C. albicans*	ACI	10 h 35 min	5 h 07 min	10 h 33 min		13 h 06 min	6 h 36 min	13 h 02 min		20 h 36 min	9 h 18 min	1 d 1 h 51 min
*C. albicans*	PSE	10 h 38 min	7 h 17 min	22 h 23 min		12 h 47 min	9 h 17 min	1 d 2 h 05 min		16 h 38 min	10 h 38 min	1 d 5 h 15 min
*C. albicans*	CNS	10 h 46 min	12 h 07 min	9 h 15 min		13 h 38 min	17 h 10 min	11 h 21 min		20 h 46 min	14 h 39 min	13 h 42 min
*C. albicans*	NDM	7 h 50 min	3 h 28 min	3 h 36 min		9 h 30 min	4 h 45 min	4 h 43 min		10 h 43 min	5 h 45 min	5 h 33 min
*C. albicans*	VIM/KPC	6 h 56 min	6 h 44 min	14 h 26 min		10 h 25 min	8 h 17 min	1 d 22 h 25 min		12 h 33 min	10 h 55 min	Negative
*C. albicans*	IMP	8 h 11 min	5 h 07min	8 h 12 min		11 h 38 min	6 h 42 min	9 h 41 min		14 h 11 min	8 h 09 min	11 h 22 min
*C. glabrata*	KPC	3 h 38 min	3 h 25 min	3 h 32 min		5 h 10 min	4 h 45 min	4 h 54 min		6 h 48 min	5 h 27 min	5 h 35 min
*N. glabratus*	MRSA	6 h 10 min	5 h 07 min	5 h 54 min		9 h 50 min	7 h 34 min	9 h 42 min		15 h 57 min	11 h 17 min	12 h 06 min
*N. glabratus*	ACI	4 h 17 min	3 h 25 min	9 h 32 min		9 h 49 min	5 h 05 min	10 h 24 min		11 h 41 min	5 h 35 min	13 h 06 min
*N. glabratus*	PSE	5 h 51 min	5 h 05 min	23 h 52 min		10 h 44 min	7 h 26 min	1 d 6 h 12 min		14 h 14 min	9 h 27 min	1 d 10 h 44 min
*N. glabratus*	CNS	5 h 38 min	5 h 46 min	6 h 23 min		10 h 31 min	10 h 17 min	10 h 34 min		12 h 36 min	9 h 57 min	13 h 40 min
*N. glabratus*	NDM	3 h 43 min	3 h 04 min	3 h 05 min		5 h 23 min	4 h 23 min	4 h 36 min		7 h 24 min	5 h 18 min	5 h 21 min
*N. glabratus*	VIM/KPC	4 h 06 min	5 h 06 min	5 h 05 min		6 h 38 min	7 h 07 min	Negative		10 h 37 min	10 h 16 min	Negative
*N. glabratus*	IMP	4 h 43 min	5 h 07 min	5 h 55 min		7 h 44 min	8 h 09 min	Negative		10 h 22 min	10 h 48 min	Negative

ATCC = Reference strain; Clinical = Clinical isolate obtained from the Microbiology Laboratory, Hospital das Clinicas, University of Sao Paulo Medical School; KPC = *Klebsiella pneumoniae* carbapenemase-producing strain (ATCC BAA 1705); MRSA = Methicillin-resistant *Staphylococcus aureus* (ATCC BAA 1026); ACI = *Acinetobacter baumannii* OXA-23; PSE = *Pseudomonas aeruginosa*; CNS = Coagulase-negative *Staphylococcus* (*S. epidermidis* ATCC 12228); NDM = *Klebsiella pneumoniae* producing New Delhi metallo-β-lactamase; VIM/KPC = *Pseudomonas aeruginosa* co-producing VIM (Verona integron-encoded metallo-β-lactamase) and KPC; IMP = *Pseudomonas aeruginosa* producing IMP-type carbapenemase.

A total of 144 bottles were used to simulate candidemia with concomitant bacteremia: 48 BD BACTEC^™^ Mycosis IC/F bottles, all with a 100% positivity rate; 48 BD BACTEC^™^ Plus Aerobic/F bottles, also all with a 100% positivity; and 48 BD BACTEC^™^ Plus Anaerobic/F bottles, with a positivity rate of 89.6% (43/48). Overall, TTD varied depending on inoculum concentration and the associated bacterial species, with some combinations detecting faster and others requiring prolonged incubation. Table 2 presents TTD values for each bottle type. Fungal recovery in BD BACTEC^™^ Mycosis IC/F, BD BACTEC^™^ Plus Aerobic/F, and BD BACTEC^™^ Plus Anaerobic/F bottles is detailed in Supplementary Tables S1, S2, and S3, respectively. Although BD BACTEC^™^ Mycosis IC/F bottles exhibited longer TTDs compared to the other bottles, culture on solid media predominantly yielded *C. albicans* and *N. glabratus*, and Gram staining confirmed the presence of both yeasts and bacteria. However, when *C. albicans* and *N. glabratus* were combined with highly resistant carbapenemase-producing bacteria such as NDM, VIM associated with KPC, or IMP, fungal recovery on agar plates was not possible, with only bacterial growth observed. Nonetheless, Gram staining still revealed both yeast and bacterial cells (Supplementary Figure S5).

## DISCUSSION

This study evaluated the performance of the BD BACTEC system in recovering a wide variety of clinically relevant fungi. In addition to *Candida* spp., species such as *C. neoformans, A. terreus, N. solani*, and *T. asahii* were included. Our results demonstrated differences in the time to detection (TTD) when comparing the growth of various *Candida* spp. in specific (BD BACTEC^™^ Mycosis IC/F) and non-specific (BD BACTEC^™^ Plus Aerobic/F and BD BACTEC^™^ Plus Anaerobic/F) blood culture bottles. These variations in time to positivity between bottle types may directly impact patient outcomes, since a 24-h delay in diagnosis can increase mortality by up to 50% (23.6%-41.4%)^12,22^. The available literature on the use of blood cultures for detecting filamentous fungi and non-*Candida* yeast species remains limited, focusing predominantly on *Candida* spp.

BD BACTEC^™^ Mycosis IC/F bottles showed clear TTD advantages for certain fungal species, particularly *C. haemuli, A. terreus, N. solani*, *N. glabratus*, and *C. albicans*, when compared with BD BACTEC^™^ Plus Aerobic/F bottles. These findings are consistent with previous studies, such as Nawrot *et al*.^23^, who showed that *N. glabratus* was detected significantly earlier in BD BACTEC^™^ Mycosis IC/F bottles (within 24 h) than in BD BACTEC™ Plus Aerobic/F bottles (approximately 48 h), a trend also reported by other authors^24,25^. Additionally, the literature suggests that *N. glabratus* exhibits relatively better growth in anaerobic media than other yeast species, although its TTD remains consistently shorter in BD BACTEC^™^ Mycosis IC/F bottles compared to standard aerobic systems. Our findings regarding *N. glabratus* and anaerobic media differ somewhat from previous reports^18,20,24^. However, this study highlights a potentially relevant role for BD BACTEC™ Plus Anaerobic/F bottles in detecting *N. glabratus* and *C. tropicalis*, the only species for which Anaerobic/F bottles successfully detected growth at all tested dilutions, with TTDs comparable to those observed in BD BACTEC^™^ Plus Aerobic/F bottles. It is important to note that *N. glabratus* is an emerging pathogen^3^ of growing clinical relevance, often associated with resistance to azoles, amphotericin B, and echinocandins, contributing to the high mortality rates linked to this species^1^.

Unlike other *Candida* species, our findings showed that *C. tropicalis* (ATCC and clinical strains) was detected earlier in aerobic media than in the specific BD BACTEC^™^ Mycosis IC/F medium. These results corroborate those of Nawrot *et al*.^23^, who also observed that *C. tropicalis* and *C. parapsilosis* were detected more rapidly in aerobic media than in BD BACTEC^™^ Mycosis IC/F, with differences ranging from 1 to 24 h. Similar findings were reported in a Brazilian study conducted in Rio Grande do Sul, in which Wilot *et al*.^26^ showed that *C. tropicalis* was detected 15 h later in BD BACTEC^™^ Mycosis IC/F.

Regarding *A. terreus*, a species capable of causing invasive infections with frequent systemic dissemination and a known tendency for resistance to amphotericin B, blood cultures often yield negative results, despite improvements in the sensitivity and speed of automated systems^27^. In our study, the BD BACTEC^™^ Mycosis IC/F bottle exhibited a shorter TTD compared to the BD BACTEC^™^ Plus Aerobic/F bottle. This result is consistent with data from Tomazin *et al*.^28^, who, when analyzing *Aspergillus* spp. at lower inoculum concentrations, observed reduced TTDs with BD BACTEC^™^ Mycosis IC/F compared to BD BACTEC™ Plus Aerobic/F bottles.

The BD BACTEC^™^ Plus Anaerobic/F bottle did not detect any of the tested filamentous fungi and exhibited a high rate of negative results for *C. auris*, *M. guilliermondii*, and *C. haemuli*. This low positivity rate may be attributed to the fact that *Candida* spp. are facultative anaerobes whose growth may be insufficient for detection under anaerobic conditions, especially due to prolonged doubling times^29^. The composition of the incubation atmosphere and medium is thus crucial for successful detection in blood cultures, as microbial growth depends directly on each species’ metabolic capacity.

Polymicrobial bloodstream infections involving bacteria and fungi are well documented in the literature^30^. This study aimed to simulate such mixed infections. In some cultures subcultured onto plates after positivity in the BACTEC FX system, yeast growth was observed exclusively in BD BACTEC^™^ Mycosis IC/F bottles, while in others, growth occurred in both BD BACTEC^™^ Mycosis IC/F and BD BACTEC^™^ Plus Aerobic/F bottles. Bacterial growth on solid media was more frequently observed in BD BACTEC^™^ Plus Aerobic/F and BD BACTEC^™^ Plus Anaerobic/F bottles.

This outcome may be related to the inhibition of bacterial growth in the BD BACTEC™ Mycosis IC/F medium, as several authors have highlighted the selective effect of its antibiotics, namely chloramphenicol and tobramycin as a key advantage for yeast recovery. Meyer *et al*.^24^ demonstrated that fungal recovery in aerobic bottles decreased significantly when bacterial co-growth occurred, with positivity dropping to 26.9%, whereas the recovery rate in BD BACTEC^™^ Mycosis IC/F bottles remained high. This highlights the superior performance of BD BACTEC^™^ Mycosis IC/F even in polymicrobial samples. In our study, however, when *C. albicans* or *N. glabratus* were combined with highly resistant bacterial strains, such as IMP-producing *P. aeruginosa*, *P. aeruginosa* co-producing VIM and KPC, and NDM-producing *K. pneumoniae*, no yeast recovery was observed on solid media from BD BACTEC™ Mycosis IC/F bottles. Although Meyer et al. did not specifically address resistance mechanisms, our findings suggest that extreme bacterial resistance may compromise fungal detection, possibly due to antagonistic interactions or altered growth kinetics. In contrast, when associated with *K. pneumoniae* and *P. aeruginosa* strains not harboring carbapenemases, bacteria already known to inhibit fungal growth both *in vitro* and *in vivo*, BD BACTEC^™^ Mycosis IC/F still enabled yeast recovery, whereas aerobic and anaerobic bottles yielded only bacterial growth. These observations support the hypothesis that bacterial resistance profiles may influence yeast recovery in BD BACTEC^™^ Mycosis IC/F bottles and warrant further investigation.

The data presented above suggest the feasibility of using different types of blood culture media in clinical practice to maximize the detection rates of candidemia and bacteremia. However, studies have shown that the cost of BD BACTEC^™^ Mycosis IC/F bottles is approximately 2.2 times higher than standard aerobic and anaerobic bottles, representing a significant limitation for low-income hospitals. In such institutions, routine use of fungus-specific media for blood cultures is already uncommon, making implementation even more challenging^31,32^.

### Limitations

Our study presents several limitations. First, the use of sterile saline instead of whole blood in the simulated candidemia model limited the biological complexity of the medium, which may influence fungal viability, growth kinetics, and time to detection. Nevertheless, this approach enabled standardized and reproducible inoculations across bottle types and was chosen due to time constraints and ethical considerations surrounding the use of blood from healthy donors. Second, only one isolate per species was tested, which does not capture the full spectrum of clinical strain variability. ATCC strains were included to provide reproducible and standardized benchmarks across bottles. Due to the limited number of available bottles, we prioritized testing emerging and multidrug-resistant species that are rarer and less studied in the literature. As a result, species such as *C. parapsilosis*, increasingly associated with nosocomial outbreaks and azole resistance, were not included, though their evaluation remains a priority for future studies. Additionally, the restricted number of blood culture bottles may have impacted the robustness of the findings. Finally, discrepancies between our results and prior studies likely reflect methodological differences, including experimental design, fungal species tested, and inoculum preparation protocols. Despite these limitations, our findings offer a valuable foundation for future research. The next step is to translate this *in vitro* pilot model into *in vivo* studies that more accurately reflect clinical conditions.

## CONCLUSION

In this *in vitro* pilot study, the BD BACTEC^™^ Mycosis IC/F bottle demonstrated a broader and slightly superior performance compared to standard aerobic and anaerobic bottles in detecting clinically relevant fungal pathogens, including *Candida* spp., filamentous fungi, and non-*Candida* yeasts. Notable advantages were observed for *N. glabratus*, *C. haemuli*, *N. solani*, and *A. terreus*, as well as in mixed fungal–bacterial infections, in which BD BACTEC^™^ Mycosis IC/F preserved fungal recovery even in the presence of multidrug-resistant bacteria. However, BD BACTEC^™^ Plus Aerobic/F bottles showed similar or even better performance for certain isolates, such as *C. tropicalis*, and remain a reliable and accessible option for the detection of most fungal pathogens in clinical settings, particularly in resource-limited environments. BD BACTEC^™^ Plus Anaerobic/F bottles demonstrated limited utility, especially for filamentous fungi and emerging yeasts. Taken together, these findings support the complementary role of BD BACTEC^™^ Mycosis IC/F bottles in fungal diagnostics, particularly in high-risk or polymicrobial contexts. Nonetheless, their higher cost and restricted availability may limit widespread implementation. Further *in vivo* studies are warranted to validate these findings and define the specific clinical scenarios in which fungal-specific blood culture bottles can provide the greatest benefit.

## Data Availability

The complete anonymized dataset supporting the findings of this study is available at https://doi.org/10.48331/SCIELODATA.UH9EH2
