# Supplementary material for: Comparative performance of BD-BACTEC® Mycosis IC/F versus standard aerobic and anaerobic bottles in simulated fungemia and mixed bloodstream infections
Source: Rev Inst Med Trop Sao Paulo. 2025 Oct 3;67:e64. doi: 10.1590/S1678-9946202567064 (PMC12499510; doi:10.1590/S1678-9946202567064)
Supplement: Supplementary file 1 [file 1678-9946-rimtsp-67-S1678-9946202567064-suppl01.pdf]

REVISTA DO INSTITUTO DE MEDICINA TROPICAL DE SÃO PAULO

JOURNAL OF THE SÃO PAULO INSTITUTE OF TROPICAL MEDICINE

SUPPLEMENTARY MATERIAL

https://doi.org/10.48331/SCIELODATA.UH9EH2

Comparative performance of BD-BACTEC® Mycosis IC/F versus standard aerobic and anaerobic bottles in simulated fungemia and mixed bloodstream infections

Juliene Carla Endo Borges[1], Lumena Pereira Machado Siqueira[1], Vera Lucia Teixeira de Freitas[2], Vítor Falcão de Oliveira[3], Adriana Satie Gonçalves Kono Magri[3], Afonso Rafael da Silva Junior[1], Evangelina da Motta Pacheco Alves de Araujo[1], Ana Paula Cury[1], Marcello Mihailenko Chaves Magri[1,3]

[1]Universidade de São Paulo, Faculdade de Medicina, Hospital das Clínicas, Divisão de Laboratório Central, Seção de Microbiologia, São Paulo, São Paulo, Brazil

[2]Universidade de São Paulo, Faculdade de Medicina, Hospital das Clínicas, Laboratório de Investigação Médica (LIM-48), São Paulo, São Paulo, Brazil

[3]Universidade de São Paulo, Faculdade de Medicina, Hospital das Clínicas, Divisão de Clínica de Moléstias Infecciosas e Parasitárias, São Paulo, São Paulo, Brazil

Correspondence to: Marcello Mihailenko Chaves Magri
Universidade de São Paulo, Faculdade de Medicina, Hospital das Clínicas, Divisão de Clínica de Moléstias Infecciosas e Parasitárias, Av. Dr. Enéas Carvalho de Aguiar, 255, sala 4028, Cerqueira César, CEP 05403-000, São Paulo, SP, Brazil
Tel: +55 11 991513561

E-mail: marcello.magri@hc.fm.usp.br

Received: 5 May 2025

Accepted: 11 August 2025

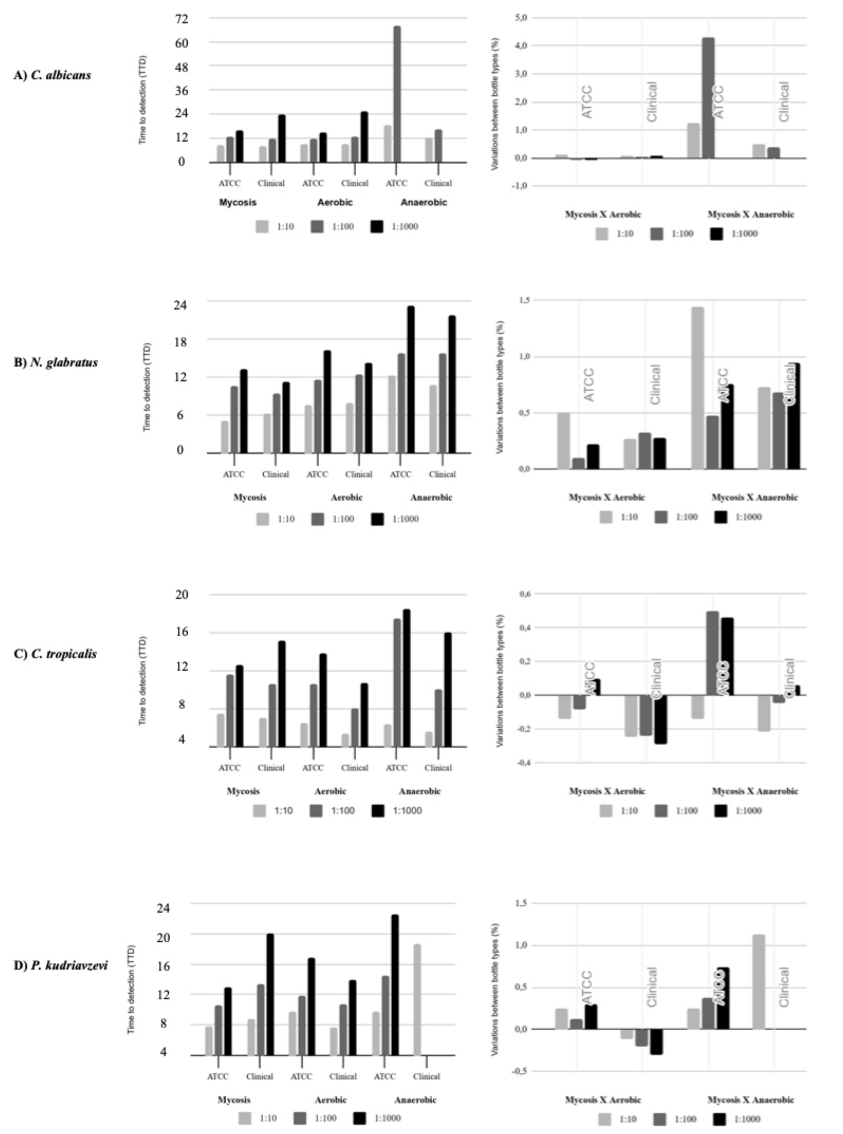

Supplementary Figure S1 - Detection performance of BD BACTEC™ Mycosis IC/F, Aerobic/F, and Anaerobic/F bottles for *Candida* species. In each panel, the left graph shows the absolute time to detection (TTD, in hours) for ATCC and clinical strains at 1:10, 1:100, and 1:1000 dilutions. The right graph depicts the percentage variation in TTD for Aerobic/F and Anaerobic/F bottles, estimated relative to Mycosis IC/F: (A-D) comparisons of TTD and percentage variation for *Candida albicans, N. glabratus, C. tropicalis*, and *Pichia kudriavzevii*. ATCC = Reference strain; Clinical = Clinical isolate obtained from the Microbiology Laboratory, Hospital das Clinicas, University of Sao Paulo Medical School.

This is an open-access article distributed under the terms of the Creative Commons Attribution License.

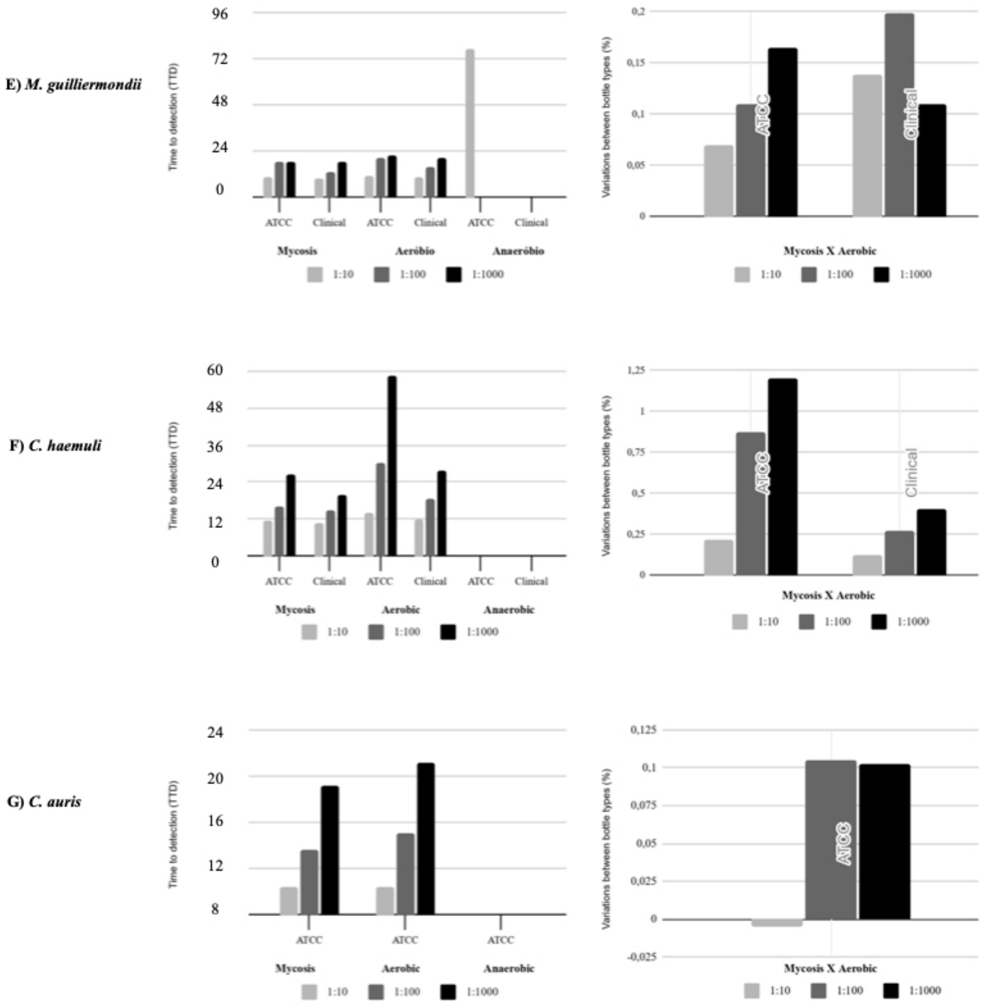

Supplementary Figure S2 - Detection performance of BD BACTEC™ Mycosis IC/F, Aerobic/F, and Anaerobic/F bottles for *Candida* species. In each panel, the left graph shows the absolute time to detection (TTD, in hours) for ATCC and clinical strains at 1:10, 1:100, and 1:1000 dilutions. The right graph depicts the percentage variation in TTD for Aerobic/F and Anaerobic/F bottles, estimated relative to Mycosis IC/F: (A–D) comparisons of TTD and percentage variation for *M. guilliermondii, C. haemuli*, and *C. auris*. ATCC = Reference strain; Clinical = Clinical isolate obtained from the Microbiology Laboratory, Hospital das Clinicas, University of Sao Paulo Medical School.

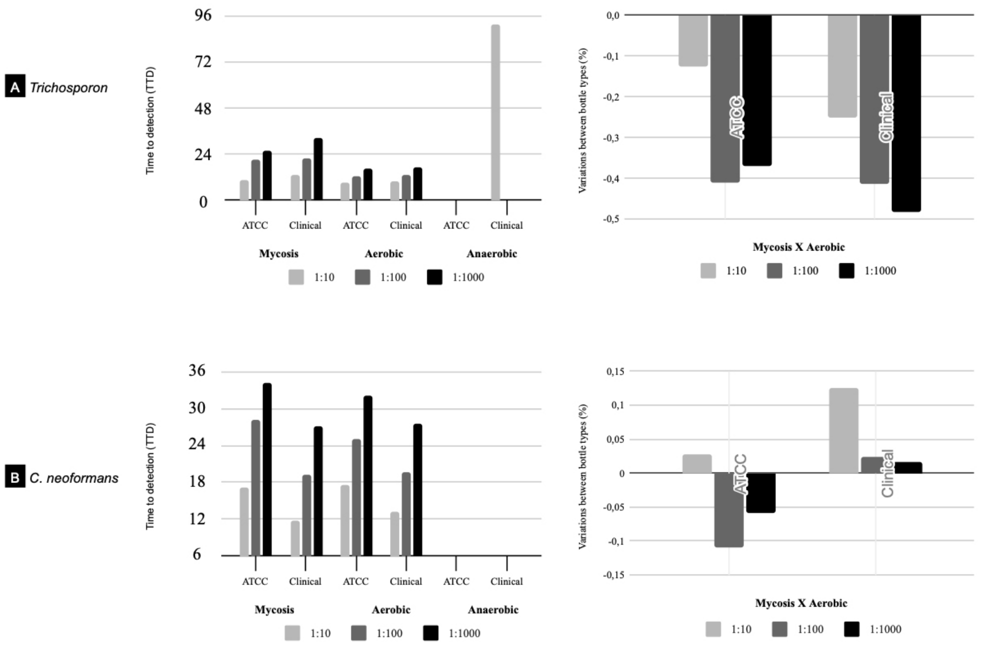

Supplementary Figure S3 - Detection performance of BD BACTEC™ Mycosis IC/F, Aerobic/F, and Anaerobic/F bottles for *Trichosporon asahii* and *Cryptococcus neoformans*. In each panel, the left graph shows the absolute time to detection (TTD, in hours) for ATCC and clinical strains at 1:10, 1:100, and 1:1000 dilutions. The right graph depicts the percentage variation in TTD for Aerobic/F and Anaerobic/F bottles, estimated relative to Mycosis IC/F. ATCC = Reference strain; Clinical = Clinical isolate obtained from the Microbiology Laboratory, Hospital das Clinicas, University of Sao Paulo Medical School.

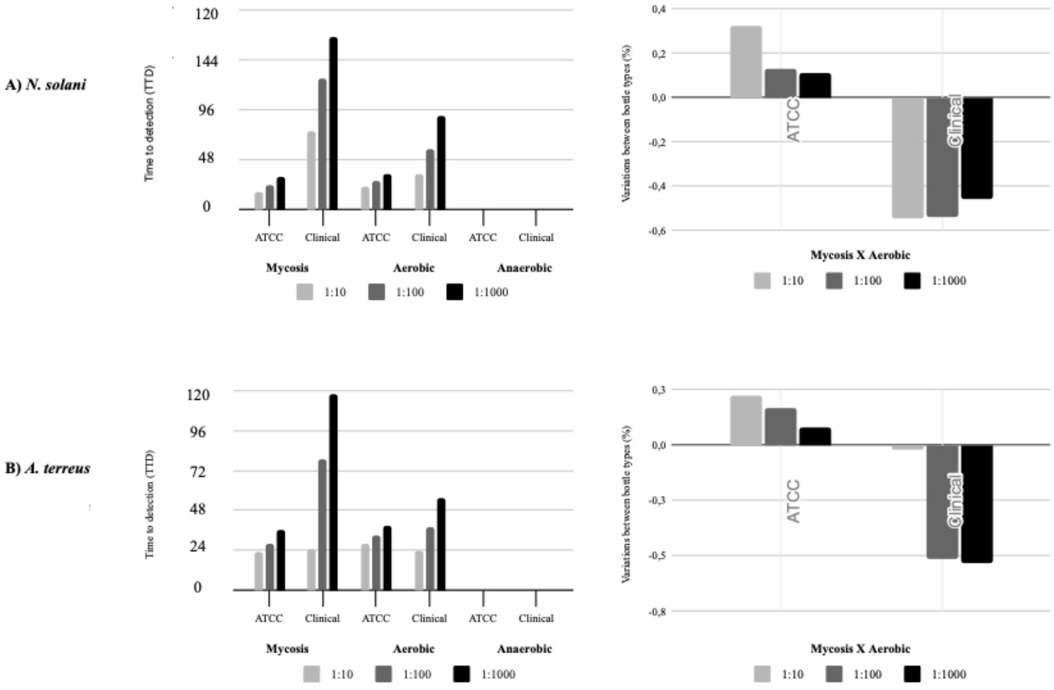

Supplementary Figure S4 - Detection performance of BD BACTEC™ Mycosis IC/F, Aerobic/F, and Anaerobic/F bottles for *Neocosmospora solani* and *Aspergillus terreus*. In each panel, the left graph shows the absolute time to detection (TTD, in hours) for ATCC and clinical strains at 1:10, 1:100, and 1:1000 dilutions. The right graph depicts the percentage variation in TTD for Aerobic/F and Anaerobic/F bottles, estimated relative to Mycosis IC/F. ATCC = Reference strain; Clinical = Clinical isolate obtained from the Microbiology Laboratory, Hospital das Clinicas, University of Sao Paulo Medical School.

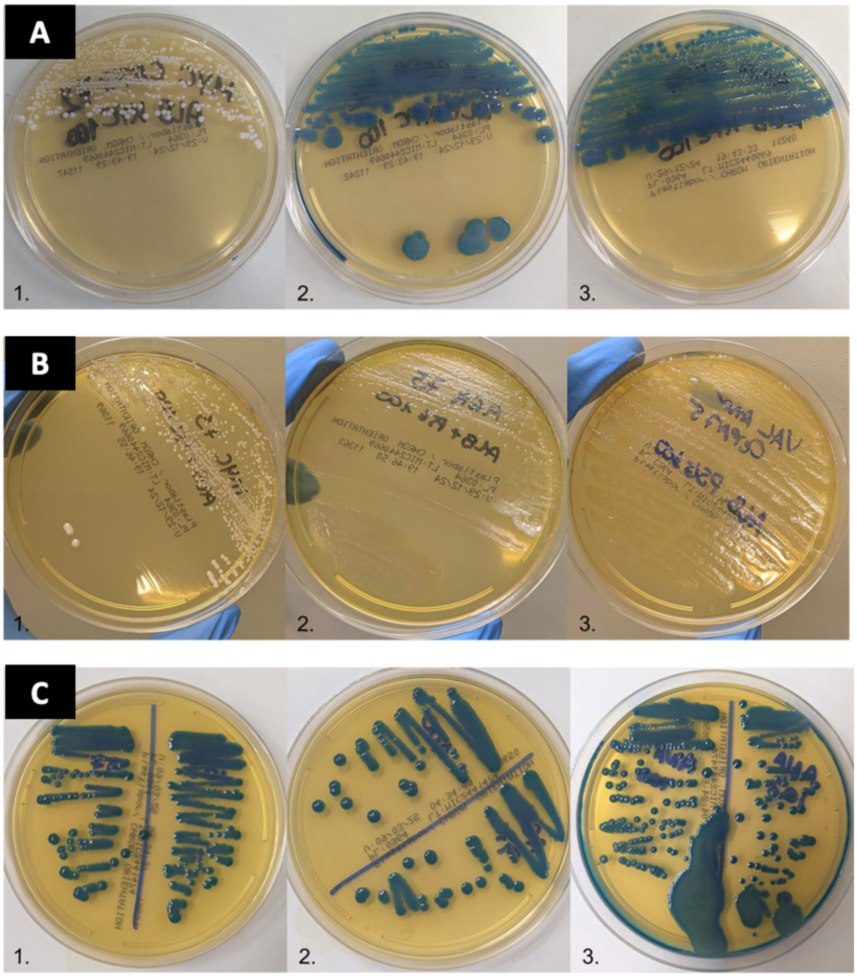

Supplementary Figure S5 - (A) Fungal recovery (CPS agar) of the combination of *C. albicans* and carbapenemase-producing *K. pneumoniae* ATCC BAA 1705 in Mycosis IC/F, Aerobic/F, and Anaerobic/F bottles, respectively. Plate 1 shows exclusive yeast growth (specific medium), whereas Plates 2 and 3 show bacterial growth (non-specific media); (B) Fungal recovery (CPS agar) of the combination of *C. albicans* and *P. aeruginosa* ATCC 2753 in Mycosis IC/F, Aerobic/F, and Anaerobic/F bottles, respectively. Plate 1 shows exclusive yeast growth (specific medium), whereas Plates 2 and 3 show bacterial growth; (C) Fungal recovery (CPS agar) of the combination of *C. albicans* and multidrug-resistant *K. pneumoniae* (NDM producer) in Mycosis IC/F (Plate 1), Aerobic/F (Plate 2), and Anaerobic/F (Plate 3). All plates show exclusive bacterial growth.

Supplementary Table S1 - Gram stain results and fungal recovery on solid media from Mycosis IC/F bottles in simulated mixed candidemia and bacteremia.

Microrganism	Dilution	GRAM MYC		Placa MYC
		1	2	
C. albicans + KPC	1:10	Y	BAC	Y
C. albicans + KPC	1:100	Y	BAC	Y
C. albicans + KPC	1:1000	Y	Y	Y
C. albicans + MRSA	1:10	Y	BAC	BAC+ Y
C. albicans + MRSA	1:100	Y	BAC	BAC+ Y
C. albicans + MRSA	1:1000	Y	Y	BAC+ Y
C. albicans + ACI	1:10	Y	BAC	Y
C. albicans + ACI	1:100	Y	BAC	Y
C. albicans + ACI	1:1000	Y	Y	Y
C. albicans + PSE	1:10	Y	BAC	Y
C. albicans + PSE	1:100	Y	BAC	Y
C. albicans + PSE	1:1000	Y	Y	Y
C. albicans + CNS	1:10	Y	BAC	Y
C. albicans + CNS	1:100	Y	BAC	Y
C. albicans + CNS	1:1000	Y	BAC	Y
C. albicans + NDM	1:10	BAC	Y	BAC
C. albicans + NDM	1:100	BAC	Y	BAC
C. albicans + NDM	1:1000	BAC	Y	BAC
C. albicans + VIM/KPC	1:10	BAC	Y	BAC+ Y
C. albicans + VIM/KPC	1:100	BAC	Y	BAC+ Y
C. albicans + VIM/KPC	1:1000	BAC	Y	BAC+ Y
C. albicans + IMP	1:10	BAC	Y	BAC
C. albicans + IMP	1:100	BAC	Y	BAC
C. albicans + IMP	1:1000	BAC	Y	BAC
N. glabratus + KPC	1:10	Y	BAC	Y
N. glabratus + KPC	1:100	Y	BAC	Y
N. glabratus + KPC	1:1000	Y	Y	Y
N. glabratus + MRSA	1:10	Y	BAC	BAC+Y
N. glabratus + MRSA	1:100	Y	BAC	BAC+Y
N. glabratus + MRSA	1:1000	Y	BAC	BAC+Y
N. glabratus + ACI	1:10	Y	BAC	Y
N. glabratus + ACI	1:100	Y	BAC	Y
N. glabratus + ACI	1:1000	Y	BAC	Y
N. glabratus + PSE	1:10	Y	BAC	Y
N. glabratus + PSE	1:100	Y	BAC	Y
N. glabratus + PSE	1:1000	Y	Y	Y
N. glabratus + CNS	1:10	Y	BAC	Y
N. glabratus + CNS	1:100	Y	BAC	Y
N. glabratus + CNS	1:1000	Y	BAC	Y
N. glabratus + NDM	1:10	BAC	Y	BAC
N. glabratus + NDM	1:100	BAC	Y	BAC
N. glabratus + NDM	1:1000	BAC	Y	BAC
N. glabratus + VIM/KPC	1:10	BAC	Y	BAC
N. glabratus + VIM/KPC	1:100	BAC	Y	BAC
N. glabratus + VIM/KPC	1:1000	BAC	Y	BAC
N. glabratus + IMP	1:10	BAC	Y	BAC
N. glabratus + IMP	1:100	BAC	Y	BAC
N. glabratus + IMP	1:1000	BAC	Y	BAC

MYC = Mycosis IC/F; KPC = *Klebsiella pneumoniae* carbapenemase-producing strain (ATCC BAA 1705); MRSA = Methicillin-resistant *Staphylococcus aureus* (ATCC BAA 1026); ACI = *Acinetobacter baumannii* OXA-23; PSE = *Pseudomonas aeruginosa*; CNS = Coagulase-negative *Staphylococcus (S. epidermidis*, ATCC 12228); NDM = *Klebsiella pneumoniae* producing New Delhi metallo-β-lactamase; VIM/KPC = *Pseudomonas aeruginosa* co-producing VIM (Verona integron-encoded metallo-β-lactamase) and KPC; IMP = *Pseudomonas aeruginosa* producing IMP-type carbapenemase; Y = Yeast; BAC = Bacteria.

Supplementary Table S2 - Gram stain results and fungal recovery on solid media from Aerobic/F bottles in simulated mixed candidemia and bacteremia.

Microrganism	Dilution	GRAM AER		Placa AER
		1	2	
C. albicans + KPC	1:10	BAC	Y	BAC
C. albicans + KPC	1:100	BAC	Y	BAC
C. albicans + KPC	1:1000	BAC	Y	BAC
C. albicans + MRSA	1:10	BAC	Y	BAC+ Y
C. albicans + MRSA	1:100	BAC	Y	BAC+ Y
C. albicans + MRSA	1:1000	BAC	Y	BAC+ Y
C. albicans + ACI	1:10	BAC	Y	BAC
C. albicans + ACI	1:100	BAC	Y	BAC
C. albicans + ACI	1:1000	BAC	Y	BAC
C. albicans + PSE	1:10	BAC	Y	BAC+ Y
C. albicans + PSE	1:100	BAC	Y	BAC+ Y
C. albicans + PSE	1:1000	BAC	Y	BAC+ Y
C. albicans + CNS	1:10	BAC	Y	BAC
C. albicans + CNS	1:100	BAC	Y	BAC
C. albicans + CNS	1:1000	BAC	Y	BAC
C. albicans + NDM	1:10	BAC	Y	BAC
C. albicans + NDM	1:100	BAC	Y	BAC
C. albicans + NDM	1:1000	BAC	Y	BAC
C. albicans + VIM/KPC	1:10	BAC	Y	BAC
C. albicans + VIM/KPC	1:100	BAC	Y	BAC
C. albicans + VIM/KPC	1:1000	BAC	Y	BAC
C. albicans + IMP	1:10	BAC	Y	BAC
C. albicans + IMP	1:100	BAC	Y	BAC
C. albicans + IMP	1:1000	BAC	Y	BAC
N. glabratus + KPC	1:10	BAC	Y	BAC
N. glabratus + KPC	1:100	BAC	Y	BAC
N. glabratus + KPC	1:1000	BAC	Y	BAC
N. glabratus + MRSA	1:10	BAC	Y	BAC+ Y
N. glabratus + MRSA	1:100	BAC	Y	BAC+ Y
N. glabratus + MRSA	1:1000	BAC	Y	BAC+ Y
N. glabratus + ACI	1:10	BAC	Y	BAC
N. glabratus + ACI	1:100	BAC	Y	BAC
N. glabratus + ACI	1:1000	BAC	Y	BAC
N. glabratus + PSE	1:10	BAC	Y	BAC+ Y
N. glabratus + PSE	1:100	BAC	Y	BAC+ Y
N. glabratus + PSE	1:1000	BAC	Y	BAC+ Y
N. glabratus + CNS	1:10	BAC	Y	Y
N. glabratus + CNS	1:100	BAC	Y	Y
N. glabratus + CNS	1:1000	BAC	Y	Y
N. glabratus + NDM	1:10	BAC	Y	BAC
N. glabratus + NDM	1:100	BAC	Y	BAC
N. glabratus + NDM	1:1000	BAC	Y	BAC
N. glabratus + VIM/KPC	1:10	BAC	Y	BAC
N. glabratus + VIM/KPC	1:100	BAC	Y	BAC
N. glabratus + VIM/KPC	1:1000	BAC	Y	BAC
N. glabratus + IMP	1:10	BAC	Y	BAC
N. glabratus + IMP	1:100	BAC	Y	BAC
N. glabratus + IMP	1:1000	BAC	Y	BAC

ERA = Aerobic/F; KPC = *Klebsiella pneumoniae* carbapenemase-producing strain (ATCC BAA 1705); MRSA = Methicillin-resistant *Staphylococcus aureus* (ATCC BAA 1026); ACI = *Acinetobacter baumannii* OXA-23; PSE = *Pseudomonas aeruginosa*; CNS = Coagulase-negative *Staphylococcus (S. epidermidis*, ATCC 12228); NDM = *Klebsiella pneumoniae* producing New Delhi metallo-β-lactamase; VIM/KPC = *Pseudomonas aeruginosa* co-producing VIM (Verona integron-encoded metallo-β-lactamase) and KPC; IMP = *Pseudomonas aeruginosa* producing IMP-type carbapenemase; Y = Yeast; BAC = Bacteria.

Supplementary Table S3 - Gram stain results and fungal recovery on solid media from Anaerobic/F bottles in simulated mixed candidemia and bacteremia.

Microrganism	Dilution	GRAM ANA		Placa ANA
		1	2	
C. albicans + KPC	1:10	BAC	Y	BAC
C. albicans + KPC	1:100	BAC	Y	BAC
C. albicans + KPC	1:1000	BAC	Y	BAC
C. albicans + MRSA	1:10	BAC	Y	BAC
C. albicans + MRSA	1:100	BAC	Y	BAC
C. albicans + MRSA	1:1000	BAC	Y	BAC
C. albicans + ACI	1:10	BAC	Y	BAC
C. albicans + ACI	1:100	BAC	Y	BAC
C. albicans + ACI	1:1000	BAC	Y	BAC
C. albicans + PSE	1:10	BAC	Y	BAC+ Y
C. albicans + PSE	1:100	BAC	Y	BAC+ Y
C. albicans + PSE	1:1000	BAC	Y	BAC
C. albicans + CNS	1:10	BAC	Y	BAC
C. albicans + CNS	1:100	BAC	Y	BAC
C. albicans + CNS	1:1000	BAC	Y	BAC
C. albicans + NDM	1:10	BAC	Y	BAC
C. albicans + NDM	1:100	BAC	Y	BAC
C. albicans + NDM	1:1000	BAC	Y	BAC
C. albicans + VIM/KPC	1:10	BAC	Y	BAC
C. albicans + VIM/KPC	1:100	BAC	Y	BAC
C. albicans + VIM/KPC	1:1000	-	Y	-
C. albicans + IMP	1:10	BAC	Y	BAC
C. albicans + IMP	1:100	BAC	Y	BAC
C. albicans + IMP	1:1000	BAC	Y	BAC
C. glabrata + KPC	1:10	BAC	Y	BAC
C. glabrata + KPC	1:100	BAC	Y	BAC
N. glabratus + KPC	1:1000	BAC	Y	BAC
N. glabratus + MRSA	1:10	BAC	Y	BAC
N. glabratus + MRSA	1:100	BAC	Y	BAC
N. glabratus + MRSA	1:1000	BAC	Y	BAC
N. glabratus + ACI	1:10	BAC	Y	BAC
N. glabratus + ACI	1:100	BAC	Y	BAC
N. glabratus + ACI	1:1000	BAC	Y	BAC
N. glabratus + PSE	1:10	BAC	Y	BAC
N. glabratus + PSE	1:100	BAC	Y	BAC
N. glabratus + PSE	1:1000	BAC	Y	BAC
N. glabratus + CNS	1:10	BAC	Y	BAC
N. glabratus + CNS	1:100	BAC	Y	BAC
N. glabratus + CNS	1:1000	BAC	Y	BAC
N. glabratus + NDM	1:10	BAC	Y	BAC
N. glabratus + NDM	1:100	BAC	Y	BAC
N. glabratus + NDM	1:1000	BAC	Y	BAC
N. glabratus + VIM/KPC	1:10	BAC	Y	BAC
N. glabratus + VIM/KPC	1:100	BAC	Y	BAC
N. glabratus + VIM/KPC	1:1000	BAC	Y	BAC
N. glabratus + IMP	1:10	BAC	Y	BAC
N. glabratus + IMP	1:100	BAC	Y	BAC
N. glabratus + IMP	1:1000	BAC	Y	BAC

ANA = Anaerobic/F; KPC = *Klebsiella pneumoniae* carbapenemase-producing strain (ATCC BAA 1705); MRSA = Methicillin-resistant *Staphylococcus aureus* (ATCC BAA 1026); ACI = *Acinetobacter baumannii* OXA-23; PSE = *Pseudomonas aeruginosa*; CNS = Coagulase-negative *Staphylococcus (S. epidermidis*, ATCC 12228); NDM = *Klebsiella pneumoniae* producing New Delhi metallo-β-lactamase; VIM/KPC = *Pseudomonas aeruginosa* co-producing VIM (Verona integron-encoded metallo-β-lactamase) and KPC; IMP = *Pseudomonas aeruginosa* producing IMP-type carbapenemase; Y = Yeast; BAC = Bacteria.
